# Biogenic Amine Contents and Microbial Characteristics of Cambodian Fermented Foods

**DOI:** 10.3390/foods9020198

**Published:** 2020-02-15

**Authors:** Dalin Ly, Sigrid Mayrhofer, Julia-Maria Schmidt, Ulrike Zitz, Konrad J. Domig

**Affiliations:** 1 Institute of Food Science, Department of Food Science and Technology, BOKU - University of Natural Resources and Life Sciences Vienna, Muthgasse 18, A-1190 Vienna, Austria; sigrid.mayrhofer@boku.ac.at (S.M.); jm_schmidt@gmx.net (J.-M.S.); ulrike.zitz@boku.ac.at (U.Z.); konrad.domig@boku.ac.at (K.J.D.); 2 Faculty of Agro-Industry, Department of Food Biotechnology, RUA - Royal University of Agriculture, Dangkor District, P.O. BOX 2696 Phnom Penh, Cambodia

**Keywords:** Cambodian fermented foods, microbial characteristics, biogenic amines, food quality, food safety

## Abstract

Naturally fermented foods are an important part of the typical diet in Cambodia. However, the food safety status of these products has not been widely studied. The aim of this study was, therefore, to provide an overview of the quality of these foods in relation to microbiology and biogenic amines. Additionally, the obtained results were compared to the habits and practices of Cambodians in handling this type of food. A total of 57 fermented foods (42 fishery and 15 vegetable products) were collected from different retail markets in the capital of Cambodia. Pathogenic *Salmonella* spp., *Listeria* spp., and *Listeria monocytogenes* were not detected in 25 g samples. Generally, less than 10^2^ cfu/g of *Staphylococcus aureus*, *Escherichia coli*, *Pseudomonas* spp., Enterobacteriaceae, and molds were present in the fermented foods. *Bacillus cereus* group members (<10^2^ to 2.3 × 10^4^ cfu/g), lactic acid bacteria (<10^2^ to 1.1 × 10^7^ cfu/g), halophilic and halotolerant bacteria (<10^2^ to 8.9 × 10^6^ cfu/g), sulfite-reducing *Clostridium* spp. (<10^2^ to 3.5 × 10^6^ cfu/g), and yeasts (<10^2^ to 1.1 × 10^6^ cfu/g) were detected in this study. Still, the presence of pathogenic and spoilage microorganisms in these fermented foods was within the acceptable ranges. Putrescine, cadaverine, tyramine, and histamine were detected in 100%, 89%, 81%, and 75% of the tested products, respectively. The concentrations of histamine (>500 ppm) and tyramine (>600 ppm) were higher than the recommended maximum levels in respectively four and one of 57 fermented foods, which represents a potential health risk. The results suggest that the production process, distribution, and domestic handling of fermented foods should be re-evaluated. Further research is needed for the establishment of applicable preservation techniques in Cambodia.

## 1. Introduction

Cambodia is an agricultural country that has a tropical climate with two distinct monsoon seasons (dry and rainy seasons). Thus, the availability of certain products is not stable through the year, and food preservation and storage are required to maintain the food supply [1]. Since food spoilage is mainly caused by microorganisms, preventing their access to susceptible foods is one method of food preservation. Another one is the inhibition of microbial growth through fermentation, salting, drying, or smoking, as it is common in Cambodia [2,3]. The storage time of food depends on factors that affect the growth of spoilage microorganisms like intrinsic food characteristics (e.g., pH, *a_w_*, composition) and extrinsic parameters (e.g., temperature, relative humidity, atmospheric gases). Due to higher ambient temperatures and moisture, food spoils faster in the tropics. As a result, it is not surprising that food security issues are reported in densely populated tropical cities [2]. However, the majority of the Cambodian population lives in rural areas where poverty is high and access to drinking water, electricity, and sanitation is limited [4]. 

Fermented foods are an important part of the typical diet in Cambodia [1]. Since Cambodia has an extensive network of waterways, freshwater fish, along with marine, fermented and preserved fish, is a major component of the diet of most Cambodians [5]. Fermented fishery products are consumed daily as main dishes, side dishes, or condiments/seasonings [5]. Additionally, they are applied as flavor enhancers due to their delicacy and high nutritional properties [6,7]. Vegetables also play an essential role in daily dishes for their nutrient content. The availability of certain fresh vegetables, however, does not last throughout the year. Depending on the varieties of domestic raw vegetables, many types of fermented vegetables have emerged in Cambodia with the popularity of traditional fermentation [8]. In the meantime, these foods have become a common part of the Cambodian diet [1,8].

Cambodian fermented foods are produced through knowledge that is passed on from generation to generation and from person to person [1]. The great majority of fermented products are locally produced by smallholders, many of them women, and sold in traditional wet markets where women also predominate as retailers [9]. Most of them are illiterate and have a poor knowledge about hygiene practices. Additionally, the awareness of food safety is limited in Cambodia. The quality of these foods is influenced by raw materials, processing methods, and climate, but there is no quality control of these determinants as well as of the finished products in Cambodia [1]. Fermented foods are generally not labelled with an appropriate shelf-life and usually stored at room temperature until they are completely consumed [1]. Fermented fishery products are usually cooked before consumption. However, fish paste and sauce can also be eaten raw and are often mixed with chili or lemon juice [1]. In contrast, fermented vegetables are normally considered as ready-to-eat (RTE) foods. As a result, it is not surprising that foodborne outbreaks are common in Cambodia [8]. But there is no coordinated food surveillance program and little analytical data regarding microbiological or chemical contamination of food are present [10]. Nevertheless, food safety is a key priority of the Cambodian government [11], and efforts to improve foodborne disease surveillance and food safety are being undertaken [10]. 

*Escherichia coli*, *Cronobacter sakazakii*, *Enterobacter* spp., opportunistic non-Enterobacteriaceae, *Staphylococcus* spp., and *Listeria* spp. have already been detected in fermented vegetables in Cambodia [8,12]. Furthermore, potentially pathogenic bacteria such as *Bacillus*, *Clostridium*, and *Staphylococcus* were found in traditional Cambodian fermented fish products [5]. Next to microbiological contamination, chemical substances can lead to acute poisoning or even long-term diseases such as cancer [13]. The most prevalent ones are biogenic amines (BAs) and biotoxins [14]. BAs are low molecular weight organic molecules, formed by microbial decarboxylation of their precursor amino acids or by transamination of aldehydes and ketones by amino acid transaminases [15]. Beside spoilage, preservative technological processes such as fermentation, salting, and ripening may increase BA formation in food. As BAs are thermostable, they cannot be inactivated by thermal treatment [13]. The most common BAs found in foods and beverages are histamine (HIS), tyramine (TYR), putrescine (PUT), and cadaverine (CAD) [16,17]. Low levels of BAs in food are not considered as a serious risk. However, when high amounts of BAs are consumed, various physiological effects may occur, namely, hypotension (in the case of HIS, PUT, and CAD) or hypertension (in the case of TYR), nausea, headache, rash, dizziness, cardiac palpitation, and even intracerebral hemorrhage and death in very severe cases [18]. BAs with more severe acute effects for human health are HIS and TYR [19]. PUT and CAD have low toxicological properties on their own, but they can act as precursor of carcinogenic *N*-nitrosamines when nitrite is present. These two BAs also potentiate the effects of HIS and TYR by inhibiting their metabolizing enzymes [19]. HIS is the only BA with regulatory limits [20]. In addition to their potential toxicity, BAs are also used to evaluate the hygienic quality of foods, as their levels in food can be an indirect indicator of excessive microbial proliferation [19]. 

Baseline surveillance data are essential to monitor the disease burden of fermented foods in Cambodia. To obtain such data, the physicochemical properties (pH, *a_w_*, and salt content) as well as the presence of certain microorganisms (spoilage and pathogenic bacteria) and the concentrations of the BAs HIS, TYR, PUT, and CAD were determined in 57 Cambodian fermented food samples within this study. The main purpose of this manuscript is to give an overview of the quality of Cambodian fermented foods, to correlate physicochemical parameters with BA contents, and to describe the prevailing habits and practices of Cambodians in dealing with this type of food.

## 2. Materials and Methods

### 2.1. Sample Collection

Fifty-seven samples of naturally fermented foods (42 raw fermented fish and 15 RTE fermented vegetable products) were randomly purchased from wet markets in Phnom Penh, the capital city of Cambodia. These products originated from various provinces of the country. Fermented fishery samples included fish sauce (teuktrey; *n* = 7), fish paste (prahok; *n* = 12), shrimp paste (kapi; *n* = 6), fermented fish (paork chav; *n* = 7; mam trey; *n* = 3)*,* sour fermented fish (paork chou; *n* = 3), and salted fish (trey proheum; *n* = 4). Fermented vegetables such as salty fermented radish (chaipov brey; *n* = 3), sweet fermented radish (chaipov paem; *n* = 3), fermented melon (trasork chav; *n* = 3), fermented mustard (spey chrourk; *n* = 3), and fermented papaya (mam lahong; *n* = 3) were bought on the next day. Detailed information about each fermented product is provided in Table 1. After purchasing, the samples were immediately packed into plastic boxes and stored at their storage temperature. One day later, the samples were transported to Vienna by airplane. At the Department of Food Science and Technology, Vienna, the samples were checked shortly after arrival and kept in their original containers at 4 °C until analysis. All samples were analyzed within the usual shelf-life of the products [21].

### 2.2. Physicochemical Properties Analysis

#### 2.2.1. Determination of pH and Water Activity (*a_w_*)

The pH value was determined by penetrating the spear tip of the Blueline 21 pH electrode (Schott AG, Mainz, Germany) into the samples. The pH values were then measured using a digital pH meter (Schott Lab 870, Mainz, Germany). 

The *a_w_* value was measured using the digital water activity meter Rotronic Hygropalm HP23-AW-A (Rotronic, Zurich, Switzerland) after equilibration at room temperature (~25 °C).

#### 2.2.2. Determination of Salt Content (NaCl)

The salt content was analyzed by potentiometric precipitation titration of chloride-ions with the 877 Titrino plus-Titrator equipped with a calomel electrode (Metrohm AG, Herisau, Switzerland). The protocol was performed according to the producer with minor modifications. Depending on the expected salt content, 1 to 10 g ± 0.01 g (a) of the samples was weighed into a glass beaker and filled with distilled water to 200 g ± 0.01 g (b). Subsequently, the samples were homogenized for 2 min at 9500 rpm using an Ultra Turrax T25 (IKA, Germany). Fifty grams ± 0.01 g (c) of the homogenized samples was weighed into a new glass beaker, and 50 mL of distilled water was added. Afterwards, 2 mL (2 M) HNO_3_ was added. The samples were then titrated with 0.1 M AgNO_3_. With the obtained results, the salt content of the original samples was calculated as % (*w/w*) NaCl = *V × M ×* 0.0584 *×* 100/m; where *V* and *M* are the volume and molarity of the AgNO_3_ standard solution used. The initial sample weight is *m*, which was calculated considering the sample preparation: *m* = *a × c/b*. The test was conducted in duplicate.

### 2.3. Microbiological Analysis

From each fermented product, a 10 g sample was aseptically collected, transferred to a stomacher bag, and homogenized (Stomacher 400 Circulator, Seward, UK) with 90 mL of buffered peptone water for 45 s at 230 rpm. Appropriate dilutions of the samples were prepared using the same diluent, and 0.1 or 1 mL aliquots of each dilution were applied on various selective media using the spread plate method or pour plate method. Lactic acid bacteria (LAB) were anaerobically grown on DeMan Rogosa Sharpe agar (MRS, Merck, Darmstadt, Germany) at 30 °C for 72 h according to ISO 15214 [22]. Enterobacteriaceae were enumerated using the pour plate method with an additional overlay on Violet Red Bile Dextrose agar (VRBD, Merck, Darmstadt, Germany) and an incubation at 37 °C for 24 h according to ISO 21528-2 [23]. *Pseudomonas* spp. were detected by plating appropriate dilutions on Cephalothin-Sodium Fusidate-Cetrimide agar (CFC, Oxoid, Hampshire, UK) and incubation at 25 °C for 44 h based on ISO 13720 [24]. Yeasts and molds were determined according to ISO 21527-2 [25] using the spread plate method on Dichloran Glycerol agar (DG18, Merck, Darmstadt, Germany). The plates were incubated at 25 °C for 5–7 d and counted on the 5th and 7th day of incubation. Selected yeast colonies were confirmed by methylene blue staining and microscopy [26]. Halophilic and halotolerant bacteria were counted after an incubation at 30 °C for 2–4 d on Tryptone Soya agar (TS, Oxoid, Hampshire, UK) supplemented with 10% NaCl (Roth, Karlsruhe, Germany) [27]. *Staphylococcus aureus* was enumerated on Baird Parker agar (BP, Merck, Darmstadt, Germany), which was incubated at 37 °C for 24 h based on ISO 6888-1/AMD 1 [28]. The plates were evaluated again after an additional 24 h incubation. The confirmation of colonies was performed using Gram-stain and DNase agar (Oxoid, Hampshire, UK) according to Kateete et al. [29]. The number of presumptive *Bacillus cereus* group members was investigated by spreading dilutions on Mannitol Yolk Polymyxin agar (MYP, Merck, Darmstadt, Germany) and incubating plates at 30 °C for 18–48 h. The evaluation was also performed after 18 h and 48 h of incubation. Colonies were confirmed by endospore staining [30]. *E. coli* was enumerated by pour plating on Tryptone Bile Glucuronic medium (TBX, Oxoid, Hampshire, UK) with an incubation at 44 °C for 18–24 h based on ISO 16649-2 [31]. The presence of sulfite-reducing *Clostridium* spp. (SRC) was analyzed by pour plating with an additional overlay on Sulfite-Cycloserin agar (SC, Oxoid, Hampshire, UK). Plates were anaerobically incubated at 37 °C for 20 h. Confirmation tests were performed using Lactose-Gelatine medium (Conda, Madrid, Spain) and Motility-Nitrate medium (Conda, Madrid, Spain) according to ISO 7937 [32]. The presence of *Salmonella* spp. was investigated using the VIDAS UP (BioMerieux, Crappone, France) *Salmonella* (SPT) system, whereas that of *Listeria* spp. and *L. monocytogenes* was tested by the VIDAS LDUO (BioMerieux, Crappone, France) system. VIDAS SPT and VIDAS LDUO are based on an enzyme-linked fluorescent immunoassay. The preparation of the samples was similar to the previous method, but 25 g of the sample was weighted in instead of 10 g. After pre-enrichment (*Listeria* spp.) and enrichment (*L. monocytogenes*, *Salmonella* spp.) steps, which were carried out according to the manufacturer’s manual, the assay steps were performed automatically by the instrument. 

### 2.4. Determination of Biogenic Amines (BAs)

The concentrations of BAs in the supernatant were analyzed by reverse-phase HPLC (Waters 2695 Separations Module, Waters, MA, USA) according to the method of Šimat et al. and Saarinen et al. [33,34]. Briefly, 1 g of the homogenized sample was extracted overnight with 5 mL of 0.4 M perchloric acid (Merk, Darmstadt, Germany). Then, the sample was centrifuged at 5000 rpm for 10 min and the supernatant was kept for further analysis. For derivatization, 80 µL of 2 M NaOH (Roth, Karlsruhe, Germany), 120 µL of saturated sodium bicarbonate solution (Merck, Darmstadt, Germany), and 400 µL of derivatization reagent (1% dansyl chloride in acetone; prepared daily; Fluka, Seelze, Germany) were added to 400 µL of sample solution. The sample was mixed and incubated for 45 min at 40 °C. Afterwards, 60 µL of 1 M ammonia solution (Roth, Karlsruhe, Germany) was added, mixed, and incubated in the dark for 60 min at room temperature. Finally, 940 µL acetonitrile (Roth, Karlsruhe, Germany) was added. The sample was mixed and centrifuged for 10 min at 13,400 rpm. A RP-18 column (Li Chro CART 250-4, 5 μm, Merck, Darmstadt, Germany) with a LiChroCART 4-4 Guard Column (RP-18, 5 µm) (Merk, Darmstadt, Germany) and a manu-CART NT cartridge holder (Merck, Darmstadt, Germany) was used for separation. The flow rate was 1 mL/min, the column temperature was 40 °C, and the injection volume was 20 µL. The mobile phase A consisted of 0.1 M ammonium acetate (Roth, Karlsruhe, Germany) and the mobile phase B was 100% acetonitrile. The following gradient was used for the separation: time = 0 min, 50% A and 50% B; time = 19 min, 10% A and 90% B; time = 20 min, 50% A and 50% B; time = 28 min, 50% A and 50% B. The detection was performed by UV–vis (Waters 2489 UV-visible detector, Waters, MA, USA) at a wavelength of 254 nm. Heptylamine (Fluka, Seelze, Germany) was used as an internal standard that was well separated from other compounds. The specificity of the method was checked using standard mixtures of 12 BA chemicals, which included spermine tetrahydrochloride, spermidine trihydrochloride, ethanolamine, isopropylamine, histamine dihydrochloride, putrescine (1,4-diaminobutan dihydrochloride), methylamine hydrochloride, agmatine sulfate, cadaverine (1,5-diaminopentan dihydrochloride), tyramine hydrochloride, dimethylamine hydrochloride, and pyrrolidine. Except for the last four chemicals, which were from Sigma-Aldrich (St. Louis, MO, USA), all chemicals were from Fluka. Standard stock solutions of BAs were prepared at 500 mg/L in 0.01 M perchloric acid. The stock solutions were diluted with 0.4 M perchloric acid to obtain series of working standard solutions (0.25, 1, 5, 10, and 15 mg/L). The derivatization procedure was the same as for the samples. All compounds were separated and could be identified by their retention times. The linearity of the method was tested by analyzing the series of working standard solutions. The correlation coefficients for the linear regression lines were better than 0.99 for all compounds. The limit of detection (LOD = 3x standard deviation of y-residuals of low concentrations/slope of calibration curve) of all BAs ranged between 0.5 and 1.5 ppm, and the limit of quantification (LOQ = 10x standard deviation of y-residuals of low concentrations/slope of calibration curve) ranged between 1.5 and 4.8 ppm. PUT, CAD, HIS, and TYR were analyzed in duplicate.

### 2.5. Statistical Analysis

Result units of quantitative microbiological analyses were cfu/g. The physicochemical parameters and BA concentrations results were analyzed with statistical analyses using the Statistical Package for the Social Sciences (SPSS, Version 20.0.0 for Windows, 2011; IBM Co., Somers, NY, USA). Data were analyzed for the degree of variation by calculating the mean and standard deviations (SDs) of the results. The significance of differences was evaluated using analysis of variance (ANOVA). A *p* value of less than 0.05 was considered statistically significant. The least-squares difference (LSD) test was used to determine the significance of differences in the physicochemical parameters and BA contents among the samples. The relationship value was determined using the Pearson correlation coefficient. 

## 3. Results

### 3.1. Physicochemical Characteristics in Fermented Foods

Physicochemical parameters such as pH, *a_w_*, and salt content were measured and compared to discuss possible causes for the different BA levels. Table 2 shows the physicochemical parameters of the tested fermented products. The pH values in fermented fishery samples were in the range of 4.4 to 7.6. Lower pH values were found in paork chav (fermented fish), while higher values were detected in kapi (shrimp paste) and trey proheum (salted fish). The *a_w_* values of the fermented fishery products ranged from 0.69 to 0.84. The lowest *a_w_* value (0.69) was detected in kapi (shrimp paste) and the highest (0.84) in paork chav (fermented fish). The salt contents were in the range of 6% to 34%, with the lowest value (6%) found in trey proheum (salted fish) and the highest (34%) in prahok (fish paste). In the fermented vegetables, the pH values were between 3.6 and 5.5. Lower pH values were found in spey chrourk (fermented mustard, 3.6–3.9) and mam lahong (fermented papaya, 3.7–3.8), while the highest value was detected in chaipov brey (salty fermented radish) (4.6). The *a_w_* values in these fermented products were between 0.75 and 0.97. The highest salt concentration (25%) was found in chaipov brey (salty fermented radish), while the lowest (2%) was detected in spey chrourk (fermented mustard) (Table 2).

Based on the statistical analysis (ANOVA) of fermented fishery products, there were significant differences (*p* < 0.05) between the physicochemical parameters of teuktrey (fish sauce) and those of paork chav (fermented fish) and trey proheum (salted fish). There was no statistically significant difference (*p* > 0.05) among the samples of teuktrey (fish sauce) and prahok (fish paste), and of paork chav (fermented fish), paork chou (sour fermented fish), and mam trey (fermented fish). Statistical analysis of fermented fish and vegetables samples was conducted separately. Regarding the fermented vegetable products, the physicochemical values of chaipov brey (salty fermented radish) were significantly different from that of spey chrourk (fermented mustard) and mam lahong (fermented papaya) (*p* < 0.05), while no significant difference was found among chaipov paem (sweet fermented radish) and trasork chav (fermented melon) (*p* > 0.05) (Table 2).

### 3.2. Presence of Microorganisms

Counts of LAB (<10^2^ to 1.1 × 10^6^ cfu/g), halophilic and halotolerant bacteria (<10^2^ to 8.9 × 10^6^ cfu/g), Enterobacteriaceae (<10^2^ cfu/g), *Pseudomonas* spp. (<10^2^ cfu/g), yeasts (<10^2^ to 1.1 × 10^6^ cfu/g), and molds (< 10^2^ to 2.3 × 10^2^ cfu/g) from the different types of fermented fish tested are indicated in Table 3. Table 3 also presents the results regarding the *B. cereus* group members (<10^2^ to 2.3 × 10^4^ cfu/g), SRC (<10^2^ to 3.5 × 10^6^ cfu/g), *S. aureus,* and *E. coli* (<10^2^ cfu/g, respectively). 

The microbial profiles found in fermented vegetables are displayed in Table 3 as well. The LAB counts were in the range of <10^2^ to 1.1 × 10^7^ cfu/g. The highest LAB counts were detected in spey chrourk (fermented mustard) and mam lahong (fermented papaya). Halophilic and halotolerant bacteria were found in numbers ranging from 2 × 10^2^ to 5.5 × 10^4^ cfu/g. The counts of *B. cereus* group members ranged from <10^2^ to 1.2 × 10^4^ cfu/g. SRC and yeasts were detected in the range of < 10^2^ to 1.5 × 10^3^ cfu/g and <10^2^ to 2.6 × 10^5^ cfu/g in the tested vegetable samples, respectively (Table 3). The counts of all other microorganisms were <10^2^ cfu/g.

### 3.3. Quantification of Biogenic Amines (BAs) in Fermented Foods

Table 4 shows the BA contents of 57 fermented product samples. The detection limits in this study were <0.5 ppm (PUT, CAD, and TYR) and <2 ppm for HIS. The results indicate that PUT was detected in quantifiable amounts in all tested fishery samples (100%), while CAD, TYR, and HIS concentrations were quantified in approximately 95%, 88%, and 86% of these products, respectively. PUT and CAD were the most frequently detected BAs in the tested samples. The highest concentrations of PUT (830 ppm), CAD (2035 ppm), HIS (840 ppm), and TYR (691 ppm) were detected in paork chav (fermented fish). PUT concentrations in 42 fishery samples were in the range between 23 to 830 ppm, with the lowest (23 ppm) presented in paork chou (sour fermented fish) and the highest (830 ppm) found in paork chav (fermented fish). The concentrations of HIS in the quantifiable fishery products (86%) ranged from 32 to 840 ppm (Table 4). The current results show that, overall, less than 50 ppm HIS was determined in all kapi (shrimp paste) samples. The concentrations of TYR in the quantifiable fishery samples (88%) ranged from 10 to 691 ppm (Table 4). In general, lower levels of TYR were detected in kapi (shrimp paste) and paork chou (sour fermented fish) than in other fermented fishery products in this study. 

The four types of BAs were also analyzed for the safety evaluation of fermented vegetables from Cambodia. The BA levels varied among the collected RTE fermented vegetables (Table 4). PUT, CAD, TYR, and HIS were detected in 100%, 73%, 60%, and 47% of the fermented vegetables, respectively. The ranges of the quantifiable BAs were from 11 to 197 ppm for PUT, 10 to 118 ppm for CAD, 18 to 103 ppm for HIS, and 7 to 86 ppm for TYR (Table 4). The results clearly show that most BA concentrations in the five types of fermented vegetables were less than 100 ppm. Even no HIS and TYR could be detected in chaipov brey (salty fermented radish) and chaipov paem (sweet fermented radish) samples (Table 4). 

According to one-way ANOVA and LSD tests of 42 fermented fisheries samples, statistically significant differences (*p* < 0.05) were found among the detected concentrations of PUT, CAD, HIS, and TYR in each product type. The statistical analysis of 15 fermented vegetable samples showed no statistical difference (*p* > 0.05) among concentrations of PUT, CAD, and HIS, while TYR concentrations were statistically different (*p* < 0.05) (Table 4).

Analyzing the correlation between total BA contents and the physicochemical parameters pH, *a_w_*, and salt content (%) in 42 fermented fishery products, a weak positive relationship between total BAs and *a_w_* values (*r* = 0.22, *p* > 0.05; *n* = 42), and a weak negative with pH values (*r* = −0.22, *p* > 0.05; *n* = 42) were found. There was no correlation among total BAs and salt content *(r* = 0.00, *p* > 0.05; *n* = 42) (Figure S1A–C). Furthermore, the linear functions between total BA contents and parameters of pH (*r* = −0.57, *p* < 0.05; *n* = 15) and salt content (*r* = −0.81, *p* < 0.05; *n* = 15) were characterized by a moderate and strong negative correlation coefficient, respectively, while a strong positive correlation between total BAs and *a_w_* value (*r* = 0.79, *p* < 0.05; *n* = 15) were determined in fermented vegetable products (Figure S2A–C). 

## 4. Discussion

### 4.1. Physicochemical Characteristics in Fermented Foods

Based on the physicochemical results, types of fermented fishery products were more different than those of fermented vegetables. Nevertheless, the pH values of this study are in good agreement with those of fermented fish products in Thailand, Vietnam, Laos, Myanmar, China, Korea, Japan, Malaysia, and Taiwan [5,35,36,37,38]. The results of the salt concentration analysis are also consistent with previous data for fermented fish products [5,38], shrimp paste [6], and fish sauce [37]. The a_w_ values of fermented fish products were comparable to fermented fish products from other countries, for example, Thai shrimp paste (0.65–0.72) and Indonesian fermented fish (0.75–0.93) [39,40]. 

The pH values found in the fermented vegetables were between 3.6 and 5.5 (Table 2). This is in agreement with a previous study, which reported that the pH of Cambodian fermented vegetables ranged from 3.6 to 6.5, depending on the raw materials and processing techniques [8]. Chaipov brey (salty fermented radish) was found to have the highest salt value (25%) of the fermented vegetables. Salty fermented radish with high salt concentrations (20–25%) has also been reported elsewhere [8]. As salt reduces *a_w_*, the lowest *a_w_* values were also determined in these samples (0.75–0.76) (Table 2).

Growth of microorganisms in foods are mainly influenced by the *a_w_* and pH [41]. The addition of salt, in turn, has an inhibitory effect on the growth of microorganisms due to its impacts on the *a_w_* value [42]. 

### 4.2. Microbiological Parameters in Fermented Foods

Microorganisms associated with fermented foods are commonly present on the external surface and in the pre- and post-harvest environment of raw materials. Additionally, they exist in the gill and gut of seafood [14].

Regarding *Bacillus* spp. and *Clostridium* spp., our results are comparable to those of Chuon et al., who also analyzed Cambodian traditional fermented fish sauce, fish paste, and shrimp paste [5]. Such traditionally home-prepared salted or fermented products are often associated with foodborne botulism [43]. However, routine testing for *C. botulinum* to ensure food safety is not recommended [43]. Instead, SRC have been proposed to identify risks from *C. botulinum* [43]. In addition to *C. botulinum*, *C. perfringens*—the most important of the SRC—poses a frequent problem and challenge in fish industry [44]. It is estimated that 10^5^ to 10^8^ CFU/g *C. perfringens* are capable of generating toxinfection [44]. Foodborne diseases that have *C. perfringens* as causative agent are related to inadequate storage, processing, and food service operations [44]. Nevertheless, no *C. perfringens* could be confirmed within this study. It is known that >10^5^ cfu/g *B. cereus* group members are potentially harmful for human consumption [45]. None of the fermented products exceeded this limit (Table 3). The survival of *B. cereus* in low numbers in several fermented products, including those based on fish and vegetables, has already been described [46]. The inactivation of this pathogen could be attributed to the presence of organic acids or higher salt concentrations [46]. Moreover, no *S. aureus* could be quantified (<10^2^ cfu/g) in the tested samples, which is in contradiction to a previous study [5]. In addition, it is reported that *S. aureus* is uniquely resistant to adverse conditions such as low *a_w_* values (0.83), high salt contents, and pH stress. Thus, most strains can grow over an *a_w_* and pH range of 0.83 to >0.99 and 4.5–9.3, respectively [47]. Although 11 of all 57 food products tested (19.3%) had an *a_w_* value in the range specified above, 10 of them (e.g., all trasork chav (fermented melon), spey chrourk (fermented mustard), and mam lahong (fermented papaya), and one chaipov paem (sweet fermented radish) product had a pH < 4.5 (Table 2). Overall, *S. aureus* could only have grown in one sample. Although *L. monocytogenes* appears to be relatively tolerant to acidic conditions, no representatives of this species as well as of other *Listeria* species were verified, which may be due to the low *a_w_* (<0.9) of most food samples tested (90%). Also, less than 10^2^ cfu/g of *Pseudomonas* spp., Enterobacteriaceae, and *E. coli* were detected in all products examined (Table 3). Furthermore, no *Salmonella* spp. could be determined using the VIDAS system. These Gram-negative bacteria are often inhibited by a salt concentration >10%, an *a_w_* value <0.95, a pH value <3.8 or >9.0 (depending on the acidulant), and the fermentation process itself [48]. LAB are not only responsible for the fermentation, they also significantly contribute to the flavor, texture, and nutritional value of fermented products [48], produce effective antimicrobial agents, and are the primary preservation factor in fermented fish products [49]. However, LAB are generally only tolerant to moderate salt concentrations (10%–18%). Consequently, their counts decrease as the salt concentration increases [50]. Forty-three samples (~75%) of all fermented products tested in this study contained more than 10% salt (Table 3). LAB were only present in high numbers in samples with less than 10% salt (Table 2 and 3). 

Since typical spoilage bacteria are generally non- or only slightly halotolerant (e.g., pseudomonads, enterobacteria), the extensive use of salt is another technological process for food preservation besides fermentation [51]. Up to 25% and 34% salinity was respectively determined for fermented vegetable and fishery products in this study. Classifying the various products according to their salt content (e.g., 0–10%, 11–20%, >20%, data not shown), the numbers of halophilic and halotolerant bacteria generally decreased with increasing salinity. 

The unfavorable conditions for bacterial growth (high salt content, a low pH or *a_w_*) may result in higher yeasts and mold numbers. These microorganisms are quite salt-tolerant [51]. As recommended by the European Food Safety Authority (EFSA), the accepted limit for molds in foods is <10^3^ cfu/g [52]. As shown in Table 3, all 57 fermented food products were acceptable regarding molds. It has been reported that <10^6^ cfu/g of yeasts are acceptable in RTE foods placed on the market [53]. An unsatisfactorily higher yeasts count (>10^6^ cfu/g) was only found in one paork chav (fermented fish) sample, which could lead to spoilage by acid and gas production [45,53]. However, the limit was just exceeded marginally (Table 3). 

According to different organizations and previous studies [45,52,53,54,55,56], the detected counts of the investigated microorganisms in this study are satisfactory. Thus, the fermented foods tested are suitable for human consumption regarding the microbiological quality.

### 4.3. Formation of BAs in Fermented Foods

A deviation in BA concentrations within a specific food category is probably due to intrinsic food characteristics such as pH and *a_w_* values, nutrients, and microbiota, as well as extrinsic factors including storage time, temperature, and manufacturing processes [57,58,59]. This may explain the wide variation of BA concentrations between the fermented fishery products and even within the same tested product type. Shalaby (1996) stated that BA levels differ not only between different food varieties but also within the same variety [57]. However, no significant difference was observed in fermented vegetables within this study.

Fish species associated with a high amount of histidine belong to the families *Scombridae, Clupeidae, Engraulidae, Coryphenidae, Pomatomidae*, and *Scombreresosidae* [60]. As seen in Table 1, the fish species of some fermented fish products belong to the families *Engraulidae* and *Scombridae* for teuktrey (fish sauce)*, Engraulidae* for paork chou (fermented fish) and *Clupeidae* for mam trey (fermented fish). Hence, these products contained higher HIS amounts (Table 4). In contrast, low HIS and TYR contents are reported in crustaceans such as shrimp [61]. Corresponding values were determined for six kapi (shrimp paste) samples within this study. Fresh fruits and vegetables such as melon, cabbage, radishes, and cucumber contain lower HIS levels; however, papaya is considered as a HIS liberator [62]. Mustard is generally an allergen, and sometimes listed as moderately high in HIS [63]. Accordingly, HIS has been found in all fermented spey chrourk (fermented mustard) and mam lahong (fermented papaya) samples, but only in one trasork chav (fermented melon) and in no chaipov (fermented radish) sample within this study. TYR has been detected in more fermented vegetable samples than HIS, although in lower concentrations. TYR and CAD have been described in few vegetables in relatively low concentrations [64]. In contrast, it has been reported that PUT is the most common BA found in food of plant origin. It is particularly abundant in vegetables [64,65] and fermented products [60]. As seen in Table 4, this BA was the only one that was verified in all fermented vegetable samples with relatively high values. 

The possible involvement of molds and yeasts in BA (especially CAD and PUT) accumulation is still discussed [19]. However, it is known that different genera, species, and strains of Gram-positive and Gram-negative bacteria are able to produce BAs by the action of microbial decarboxylases [66]. In particular, Enterobacteriaceae were identified as HIS-producing bacteria, but also halophilic and halotolerant bacteria (among other representatives of the families Enterobacteriaceae, Pseudomonadaceae, and the genera *Photobacterium*, *Vibrio*, and *Staphylococcus*), LAB, *Bacillus* spp., and *Clostridium* spp. were said to be capable of HIS formation [14,57,67]. According to Rodriguez-Jerez et al., microbial species with the capacity to form HIS and those with the capacity to form other BAs are similar [68]. Thus, Enterobacteriaceae were also reported to produce PUT, CAD, and to a lesser extent TYR. These BAs have also been detected when testing various *Bacillus* strains [67]. However, TYR should be mainly formed by LAB (*Lactobacillus*, *Enterococcus*) during fermentation [16]. Next to strains of the genera *Clostridium*, *Pseudomonas,* and *Staphylococcus*, LAB (*Enterococcus*, *Lactococcus*) are also involved in the production of PUT. It should be kept in mind that decarboxylase activities are often related to strains rather than to species or genera [69]. The capabilities of such strains, in turn, vary depending on the type and even batch of food product from which the strains are isolated [67].

The main factors affecting microbial activities in food are temperature, salt concentration, and pH [19]. Most fermented foods in this study had a pH value within the range of 3.0 to 6.0 (79%, Table 2), providing an acidic environment. The transcription of many decarboxylase genes is induced by a low pH value, which improves the fitness of the microbial cells subjected to acidic stress [19]. As the decarboxylation of amino acids is a mechanism of BA-forming bacteria to counteract acidic stress and to adapt to environmental conditions, their decarboxylase activity increases, resulting in higher BA concentrations [7,58]. Hence, it contributed to higher BA contents (Table 4). This effect could be confirmed within this study, as weak and moderate negative correlations (*r* = −0.22 and −0.57) were respectively found between the total BA contents and pH values for fermented fishery and fermented vegetable products. A strong negative linear fit (*r* = −0.81) could be detected between total BAs and salt content in fermented vegetables, whereas there was no correlation between these parameters in fermented fishery products (Figures S1 and S2). In general, increasing salt concentrations contribute to the reduction of BA accumulation in foods, mainly reducing the metabolic activities of decarboxylase-positive microorganisms [19] as it may have been the case for the fermented vegetables. However, a possible enhancing effect of NaCl on the BA production has also been described [19]. Thus, stressed cells seem to activate the decarboxylating pathways in the framework of more complex response systems [19] being probably more present in fermented fishery than vegetable products. In contrast, the rate of BA accumulation decreases with the decrease of *a_w_* values due to the water loss [19]. Correspondingly, a positive correlation should be observed between total BA contents and *a_w_* values. In fact, weak and strong positive relationships were determined for fermented fishery (*r* = 0.22) and vegetable (*r* = 0.79) products, respectively. 

The ability of microorganisms to produce BAs is limited by low temperature [19]. Within this study, samples were stored at 4°C until investigation. However, fermented fishery and vegetable products are usually stored at room temperature in Cambodia due to the given conditions. Thus, even higher BA amounts could be expected for these products in this country. Paork chav (fermented fish) and other fermented fish products are stored at room temperature up to a year [21]. In the case of fermented vegetables, the salt content seems to be particularly relevant for the storage time. Thus, vegetables with 5–6% salt should be sold as soon as possible, while chaipov brey (salty fermented radish) samples with high salt concentrations (20–25%) have a longer storage time [8]. In this regard, higher BA values were detected in fermented vegetables with lower salt contents (2–5%).

### 4.4. BAs and Food Safety 

Table 5 shows the distribution of the tested fermented food products according to the different allowable limits. Several organizations have set legal maximum limits on HIS concentrations in fermented foods that should ensure safe human consumption if the limits are not exceeded. Such organizations are the US Food and Drug Administration (FDA) with 50 ppm, FAO/WHO with 200 ppm for fish and fishery products, respectively, and EFSA with 400 ppm for fishery products that have undergone enzyme maturation treatment in brine [60,70,71]. The HIS level in fish sauce has been regulated in particular by the Codex Alimentarius Commission (CAC) and EFSA, with a maximum allowable limit of 400 ppm [55,72]. Correspondingly, the contents of HIS in all teuktrey (fish sauce) products did not exceed 400 ppm (Table 5). Thus, the levels of HIS in teuktrey (fish sauce) products in the current study can be regarded as safe for human consumption according to EFSA and CAC. Due to numerous outbreaks with toxic HIS concentrations ≥500 ppm [60], one paork chou (sour fermented fish), one mam trey (fermented fish), and two paork chav (fermented fish) products, representing about 7% (4/57) of the fermented products (Table 5), could pose a health risk. Although a food safety criterion is only set for HIS, HIS is not the only BA responsible for health hazards. Healthy individuals should also not be exposed to TYR values of 600 ppm or more by meal as recommended by EFSA [60]. The concentrations of TYR in all tested products were less than 600 ppm (Table 4), except for one paork chav (fermented fish) sample, which may constitute a health hazard [60]. Nevertheless, this sample is still fine according to Prester et al., who suggested a dietary value of up to 800 ppm of TYR as acceptable. More than 1080 ppm are toxic for adults [61]. 

TYR concentrations from <0.4 to 270.6 ppm in commercially Chinese fish sauces [73], from 77.5 to 381.1 ppm in Korean anchovy sauces [74], and from 0 to 1178 ppm in commercial fish sauces the Far East sold at German markets [75] were reported. Hence, the concentrations of TYR in teuktrey (fish sauce) from retail markets in Cambodia were generally within or even below these concentrations (Table 4). 

It has also been reported that the acute toxicity levels of TYR and CAD are respectively greater than 2000 ppm and the oral toxicity level of PUT is 2000 ppm [15]. It is known that TYR has a stronger and more rapid cytotoxic effect than HIS [76]. Unlike HIS and TYR, the pharmacological activities of PUT and CAD seem to be less potent. Nonetheless, both amines show in vitro cytotoxicity at concentrations easily reached in inherently BA-rich foods [77] and enhance the toxicity of HIS and TYR [19]. In the tested teuktrey (fish sauce) products, the highest levels of PUT (404 ppm) and CAD (766 ppm) were higher than those in fish sauce sold at Malaysian markets (242.8 ppm PUT and 704.7 ppm CAD) [78] and Chinese markets (276.6 ppm PUT and 606.3 ppm CAD) [73] but much lower than the levels in imported fish sauce products sold at German markets (1257 ppm PUT and 1429 ppm CAD) [75], Austrian markets (510 ppm PUT and 1540 ppm CAD) [65], and other European markets (1220 ppm PUT and 1150 ppm CAD) [60]. Extremely high PUT and CAD contents characterize inferior fish sauces, which may be due to the minor production hygiene, less salt content (<20%), the type of fish species, and storage condition [19,61]. Nevertheless, a health risk from consuming such a fish sauce is likely to be excluded due to the relatively small average intake [75]. Interestingly, the PUT concentrations of fish sauce samples were generally lower than the associated CAD concentrations (Table 4). The complexity of fish protein, which releases more lysine (precursor of CAD) during the fermentation of fish sauce, resulting in increased CAD concentrations could be the reason [78]. The current results also show that the highest PUT and CAD values in fish and shrimp pastes were higher than those in paste products in Taiwan [35] and in the Maldives [79]. Generally, BAs were detected in low levels in the tested fermented vegetables, which should not cause any risk when consumed. These results were consistent with previous studies [60,80], which reported that fermented vegetables should be considered as low-risk products in terms of BAs.

### 4.5. BAs and Food Quality

The HIS content alone may be a reliable indicator of food safety, but not of food quality. TYR and CAD are used as spoilage index [81]. Other authors have considered PUT and CAD as spoilage indicators [82]. Furthermore, PUT and CAD increase with longer storage times [19] and give strong unpleasant decaying odors at very low concentrations [75]. Therefore, the PUT and CAD concentration could be used as quality indicator [19], and their accumulation should be avoided [15,77]. These BAs are also included in the biogenic amine index (BAI) [83]. The BAI, the sum of HIS, TYR, PUT, and CAD, is more indicative of food quality, as these BAs are mostly produced at the end of shelf-life, indicating spoilage [83,84]. This index was also established to facilitate the evaluation and comparison of the BA concentrations in food. However, the usefulness of the BAI as quality index depends on many factors, mainly concerning the nature of the product (e.g., fresh or fermented food). Owing to the number of different factors (e.g., fermentation, maturation, starters), BA amounts vary much more in fermented products [13]. Thus, the BAI has proven to be more satisfactory for fresh products, and there is a BAI for freshwater fish of 50 ppm [85], while it is missing for fermented fishery products. The only BAI for a fermented food product was given by Wortberg and Woller [84] for Bologna sausages at 500 ppm. The higher BAI mainly results from the fermentation process and/or ripening. Using this limit, about one-third (31%) of the fermented fishery products in this study had a BAI of less than 500 ppm, while two-thirds (69%) had a higher BAI (>500 ppm), indicating a poor hygienic quality (Figure S3). Of the fermented vegetables, about 13% (2/15) had a BAI higher than 300 ppm (Figure S4). This value corresponds to the sum of HIS, TYR, PUT, and CAD, which should not be exceeded by acceptable sauerkraut [57]. 

In view of these results, the production process, distribution, and domestic handling of fermented products should be re-evaluated under strict hygienic practices together with the hazard analysis critical control point (HACCP) approach in order to minimize the content of BAs and microbiological contamination. The storage of food by cooling or freezing requires electricity that is not available to all Cambodians. Therefore, preservation techniques, such as the use of antimicrobial substances and/or autochthonous starter cultures, which are characterized by the absence of any BA formation ability or the presence of a BA detoxification activity, should be tested for their possible application on an industrial and small scale. 

## 5. Conclusions

The presence of microorganisms in the examined fermented samples presented no health risk since pathogenic and spoilage microorganisms were in acceptable ranges. Nevertheless, one paork chou (sour fermented fish), one mam trey (fermented fish), and two paork chav (fermented fish) products represent a health risk because of the high level of HIS (>500 ppm). One of the paork chav (fermented fish) samples additionally exceeded the recommended TYR maximum (>600 ppm) per meal. The totals of all BAs tested were higher than the recommended corresponding BAI values in about 69% of the tested fermented fishery and 13% of the vegetable products. This may indicate a poor hygienic quality for these products. Hence, the production process, distribution, and domestic handling of fermented products in Cambodia should be re-evaluated in order to minimize the content of BAs and microbiological contamination. Further research is required to establish preservation techniques that could be applied on an industrial and small-scale in Cambodia. 

## Figures and Tables

**Table 1:** Detailed information of fermented food products.

Product Type	English Name	Local Name of Fish/Vegetable Species ^a^	Scientific Family Name of Fish/Vegetable Species	Major Ingredients	Usage	Market Origin
**Fermented Fishery Products**						
Teuktrey (*n* = 7)	Fish sauce	Trey Kakeum	*Engraulidae*	Freshwater/sea fish, salt	Side dish, Condiment, Seasoning	Chamkadaung, Oreusey
		Trey Kamong	*Scombridae*			
		Trey Riel	*Cyprinidae*			
		Trey Kanchanhchras	*Ambassis*			
		Trey Linh	*Cyprinidae*			
		Trey Kralong	*Cyprinidae*			
		Trey Achkok	*Cyprinidae*			
Prahok (*n* = 12)	Fish paste	Trey Riel	*Cyprinidae*	Freshwater fish, salt	Main dish, Side dish, Condiment, Seasoning	Deumkor, Oreusey, Chamkadaung
		Trey Chhkork	*Cyprinidae*			
		Trey Ptourk	*Channidae*			
		Trey Kampleanh	*Osphronemidae*			
Kapi (*n* = 6)	Shrimp paste	-	-	Tiny marine shrimp, salt	Side dish, Condiment, Seasoning	Kandal, Chas, Oreusey
Paork chav (*n* = 7)	Fermented fish	Trey Por	*Pangasiidae*	Freshwater fish, brown glutinous rice, salt	Main dish, Side dish	Thmey, Oreusey
		Trey Pra	*Pangasiidae*			
		Trey Chhkork	*Cyprinidae*			
Paork chou (*n* = 3)	Sour fermented fish	Trey Sleuk Reusey	*Engraulidae*	Freshwater fish, rice, salt	Main dish, Side dish	Chamkadaung, Oreusey
Mam trey (*n* = 3)	Fermented fish	Trey Bondol Ampov	*Clupeidae*	Freshwater fish, palm sugar, salt	Main dish, Side dish	Thmey
Trey proheum (*n* = 4)	Salted fish	Trey Pra, Trey Proma	*Pangasiidae*, *Sciaenidae,*	Freshwater fish, salt	Main dish, Seasoning	Thmey
**Fermented Vegetables**						
Chaipov brey (*n* = 3)	Salty fermented radish	Chaitav	*Brassicaceae*	Chinese white radish, salt	Side dish, Seasoning, Appetizer	Kandal Chas Oreusey
Chaipov paem (*n* = 3)	Sweet fermented radish	Chaitav	*Brassicaceae*	Chinese white radish, sugar	Main dish, Side dish, Appetizer	Kandal Chas Oreusey
Trasork chav (*n* = 3)	Fermented melon	Trasork	*Cucurbitaceae*	Baby melon, salt, purple sticky rice	Side dish	Thmey
Spey chrourk (*n* = 3)	Fermented mustard	Speythom	*Brassicaceae*	Chinese mustard, salt	Side dish	Phumreusey Limcheanghak
Mam lahong (*n* = 3)	Fermented papaya	Lahong	*Caricaceae*	Green papaya, tiny fermented fish, salt, herb	Side dish	Phumreusey Limcheanghak

^a^ Name in Cambodian.

**Table 2:** Physicochemical characteristics (pH, *a_w_*, % NaCl) of fermented food products.

Product Types	English Name	Physicochemical Characteristics (Mean ± SD)		
		pH	*a_w_*	Salt Content (%)
**Fermented Fishery Products**				
Teuktrey (*n* = 7)	Fish sauce	4.8–6.3 ^≠^ (5.5 ± 0.6 ^¥^) ^a^	0.72–0.82 (0.76 ± 0.04) ^a^	19–25 (22 ± 2) ^a^
Prahok (*n* = 12)	Fish paste	5.3–5.7 (5.5 ± 0.1) ^a^	0.71–0.80 (0.73 ± 0.02) ^ab^	15–34 (21 ± 5) ^a^
Kapi (*n* = 6)	Shrimp paste	6.6–7.3 (7.0 ± 0.2) ^b^	0.69–0.72 (0.71 ± 0.01) ^b^	13–28 (19 ± 7) ^a^
Paork chav (*n* = 7)	Fermented fish	4.4–5.1 (4.8 ± 0.3) ^c^	0.74–0.84 (0.80 ± 0.03) ^c^	9–14 (11 ± 2) ^b^
Paork chou (*n* = 3)	Sour fermented fish	4.9–5.1 (5.0 ± 0.1) ^c^	0.74–0.76 (0.75 ± 0.01) ^a^	13–15 (14 ± 1) ^b^
Mam trey (*n* = 3)	Fermented fish	4.9–5.6 (5.3 ± 0.4) ^ac^	0.76–0.78 (0.77 ± 0.01) ^ac^	8–14 (10 ± 3) ^b^
Trey proheum (*n* = 4)	Salted fish	6.3–7.6 (6.8 ± 0.6) ^b^	0.78–0.83 (0.80 ± 0.02) ^c^	6–17 (12 ± 5) ^b^
**Fermented Vegetables**				
Chaipov brey (*n* = 3)	Salty fermented radish	4.4–4.8 (4.6 ± 0.2) ^g^	0.75–0.76 (0.75 ± 0.01) ^g^	24–25 (24 ± 0.4) ^g^
Chaipov paem (*n* = 3)	Sweet fermented radish	3.9–5.5 (4.5 ± 0.9) ^gh^	0.78–0.87 (0.82 ± 0.04) ^h^	10–18 (13 ± 4) ^h^
Trasork chav (*n* = 3)	Fermented melon	4.1–4.4 (4.3 ± 0.2) ^gh^	0.76–0.83 (0.80 ± 0.03) ^h^	9–13 (10 ± 2) ^h^
Spey chrourk (*n* = 3)	Fermented mustard	3.6–3.9 (3.7 ± 0.2) ^h^	0.96–0.97 (0.97 ± 0.01) ^i^	2–5 (4 ± 1) ^i^
Mam lahong (*n* = 3)	Fermented papaya	3.7–3.8 (3.7 ± 0.1) ^h^	0.91–0.94 (0.92 ± 0.01) ^i^	3–4 (3 ± 1) ^i^

^≠^ Ranged values (minimum to maximum). ^¥^ Mean ± SD (standard deviation). Values with different superscript letters in the same column indicate significant differences at *p* < 0.05 by LSD test. Statistical analysis of fermented fish and vegetable samples was conducted separately.

**Table 3:** Microbial profiles found in fermented food products.

Product Type	English Name	Microorganisms (cfu/g)									
		LAB ^a^	Halophilic & Halotolerant Bacteria	Entero-Bacteriaceae	*Pseudomonas* spp.	Yeasts	Molds	*B. cereus* Group Members	SRC ^b^	*S. aureus*	*E. coli*
**Fermented Fishery Products**											
Teuktrey (*n* = 7)	Fish sauce	<10^2^	<10^2^	<10^2^	<10^2^	<10^2^	<10^2^	<10^2^	<10^2^	<10^2^	<10^2^
Prahok (*n* = 12)	Fish paste	<10^2^–1.5 × 10^2^	10^2^–9.5 × 10^3^	<10^2^	<10^2^	<10^2^	<10^2^	<10^2^–1.6 × 10^3^	2.4 × 10^2^–2.8 × 10^6^	<10^2^	<10^2^
Kapi (*n* = 6)	Shrimp paste	<10^2^–2.8 × 10^3^	5.2 × 10^3^–5 × 10^5^	<10^2^	<10^2^	<10^2^	<10^2^	<10^2^–6.8 × 10^3^	2 × 10^2^–1.3 × 10^5^	<10^2^	<10^2^
Paork chav (*n* = 7)	Fermented fish	<10^2^–1.6 × 10^3^	1.2 × 10^3^–2.6 × 10^5^	<10^2^	<10^2^	<10^2^–1.1 × 10^6^	<10^2^	10^2^–2.3 × 10^4^	<10^2^–9 × 10^4^	<10^2^	<10^2^
Paork chou (*n* = 3)	Sour fermented fish	<10^2^	2 × 10^2^–9 × 10^3^	<10^2^	<10^2^	<10^2^–2.7 × 10^4^	<10^2^	<10^2^–3.5 × 10^3^	4 × 10^2^–1.2 × 10^4^	<10^2^	<10^2^
Mam trey (*n* = 3)	Fermented fish	<10^2^–1.2 × 10^3^	10^3^–5.1 × 10^4^	<10^2^	<10^2^	<10^2^	<10^2^	1.1 × 10^2^–1.2 × 10^4^	1.9 × 10^3^–3.5 × 10^6^	<10^2^	<10^2^
Trey proheum (*n* = 4)	Salted fish	<10^2^–1.1 × 10^6^	2.3 × 10^4^–8.9 × 10^6^	<10^2^	<10^2^	<10^2^	<10^2^	<10^2^–2.6 × 10^2^	<10^2^–1.6 × 10^5^	<10^2^	<10^2^
**Fermented Vegetables**											
Chaipov brey (*n* = 3)	Salty fermented radish	<10^2^	3 × 10^3^–1.6 × 10^4^	<10^2^	<10^2^	<10^2^	<10^2^	<10^2^	<10^2^	<10^2^	<10^2^
Chaipov paem (*n* = 3)	Sweet fermented radish	<10^2^	1.7 × 10^4^–5.5 × 10^4^	<10^2^	<10^2^	<10^2^	<10^2^	<10^2^–2.2 × 10^2^	<10^2^–3 × 10^2^	<10^2^	<10^2^
Trasork chav (*n* = 3)	Fermented melon	<10^2^	2 × 10^2^–8 × 10^3^	<10^2^	<10^2^	<10^2^	<10^2^	<10^2^	<10^2^	<10^2^	<10^2^
Spey chrourk (*n* = 3)	Fermented mustard	<10^2^–1.1 × 10^7^	7.3 × 10^2^–2 × 10^4^	<10^2^	<10^2^	<10^2^–2.6 × 10^3^	<10^2^	2 × 10^2^–1.2 × 10^4^	<10^2^	<10^2^	<10^2^
Mam lahong (*n* = 3)	Fermented papaya	<10^2^–5.8 × 10^6^	2 × 10^2^–1.1 × 10^3^	<10^2^	<10^2^	1.7 × 10^2^–2.6 × 10^5^	<10^2^	<10^2^–1.6 × 10^2^	3.2 × 10^2^–1.5 × 10^3^	<10^2^	<10^2^

^a^ Lactic acid bacteria; ^b^ sulfite-reducing clostridia.

**Table 4:** Contents of biogenic amines in fermented food products.

Product Type	English Name	Biogenic Amines (ppm)			
		PUT *	CAD	HIS	TYR
**Fermented Fishery Products**					
Teuktrey (*n* = 7)	Fish sauce	75–404 ^≠^ (233 ± 126 ^¥^) ^ab^	99–766 (368 ± 231) ^abc^	40–253 (155 ± 74) ^ab^	39–342 (144 ± 110) ^ab^
Prahok (*n* = 12)	Fish paste	191–649 (360 ± 150) ^b^	119–899 (522 ± 231) ^abc^	35–408 (179 ± 115) ^abc^	76–594 (218 ± 159) ^ab^
Kapi (*n* = 6)	Shrimp paste	29–294 (112 ± 99) ^ac^	ND ^#^–270	ND–46	ND–57
Paork chav (*n* = 7)	Fermented fish	33–830 (386 ± 337) ^b^	38–2035 (672 ± 782) ^bc^	32–840 (422 ± 264) ^c^	10–691 (299 ± 294) ^b^
Paork chou (*n* = 3)	Sour fermented fish	23–92 (49 ± 37) ^a^	26–69 (43 ± 24) ^a^	46–559 (260 ± 267) ^bc^	ND–82
Mam trey (*n* = 3)	Fermented fish	37–569 (378 ± 296) ^bc^	23–1470 (930 ± 788) ^c^	33–732 (320 ± 366) ^bc^	ND–196
Trey proheum (*n* = 4)	Salted fish	72–278 (153 ± 90) ^ab^	187–485 (297 ± 130) ^ab^	ND–183	53–118 (79 ± 30) ^a^
**Fermented Vegetables**					
Chaipov brey (*n* = 3)	Salty fermented radish	12–18 (15 ± 3) ^g^	ND–12	ND	ND
Chaipov paem (*n* = 3)	Sweet fermented radish	11–16 (14 ± 3) ^g^	ND–10	ND	ND
Trasork chav (*n* = 3)	Fermented melon	28–107 (70 ± 40) ^g^	ND–23	ND–18	7–30 (15 ± 13) ^g^
Spey chrourk (*n* = 3)	Fermented mustard	33–197 (95 ± 89) ^g^	16–51 (29 ± 19) ^g^	34–103 (66 ± 35) ^g^	22–86 (44 ± 36) ^gh^
Mam lahong (*n* = 3)	Fermented papaya	72–184 (119 ± 58) ^g^	22–118 (68 ± 48) ^g^	33–72 (49 ± 20) ^g^	38–63 (53 ± 13) ^h^

* PUT, putrescine; CAD, cadaverine; HIS, histamine; TYR, tyramine; ^≠^ Ranged values (minimum to maximum) ^¥^ Mean ± SD; ^#^ ND, not detected (Limit of detection < 0.5 ppm for PUT, CAD, and TYR; <2 ppm for HIS). Values with different superscript letters in the same column indicate significant differences at *p* < 0.05 by LSD test. Statistical analysis of fermented fish and vegetable samples was conducted separately.

**Table 5:** Distribution of fermented foods with quantifiable histamine contents.

Product Type	English Name	Histamine Contents (ppm)					
		≤50	>50 to 200	>200 to 400	>400 to <500	≥500	Total
**Fermented Fishery Products—Number of Quantifiable Samples**							
Teuktrey (*n* = 7)	Fish sauce	1 (14%)	3 (43%)	3 (43%)			7
Prahok (*n* = 12)	Fish paste	1 (9%)	7 (58%)	3 (25%)	1 (8%)		12
Kapi (*n* = 6)	Shrimp paste	6 (100%)					6
Paork chav (*n* = 7)	Fermented fish	1 (14%)		2 (29%)	2 (29%)	2 (29%)	7
Paork chou (*n* = 3)	Sour fermented fish	1 (33%)	1 (33%)			1 (33%)	3
Mam trey (*n* = 3)	Fermented fish	1 (33%)	1 (33%)			1 (33%)	3
Trey proheum (*n* = 4)	Salted fish	2 (50%)	2 (50%)				4
Total		13 (31%)	14 (33%)	8 (19%)	3 (7%)	4 (10%)	42 (100%)
**Fermented Vegetables—Number of Quantifiable Samples**							
Chaipov brey (*n* = 3)	Salty fermented radish	3 (100%)					3
Chaipov paem (*n* = 3)	Sweet fermented radish	3 (100%)					3
Trasork chav (*n* = 3)	Fermented melon	3 (100%)					3
Spey chrourk (*n* = 3)	Fermented mustard	1 (33%)	2 (67%)				3
Mam lahong (*n* = 3)	Fermented papaya	2 (67%)	1 (33%)				3
Total		12 (80%)	3 (20%)				15 (100%)

## Supplementary Materials

The following are available online at https://www.mdpi.com/2304-8158/9/2/198/s1, Figure S1: Linear fitting between total biogenic amines and physicochemical parameters, including pH (A), water activity (B), and salt content (C), in fermented fishery products (*n* = 42). Each dot indicates a data set obtained from a single sample, Figure S2. Linear fitting between total biogenic amines and physicochemical parameters, including pH (A), water activity (B), and salt content (C), in fermented vegetable products (*n* = 15). Each dot indicates a data set obtained from a single sample, Figure S3. Biogenic amine index (BAI) evaluated for 42 samples of fermented fishery products. The bold horizontal line describes the limit value of 500 ppm, which is used to distinguish between fermented fishery products of good and poor hygienic quality, Figure S4. Biogenic amine index (BAI) evaluated for 15 samples of fermented vegetable products. The bold horizontal line describes the limit value of 300 ppm, which is used to distinguish between fermented vegetable products of good and poor hygienic quality.
