# Supplementary material for: Biogenic Amine Contents and Microbial Characteristics of Cambodian Fermented Foods
Source: Foods. 2020 Feb 15;9(2):198. doi: 10.3390/foods9020198 (PMC7074300; doi:10.3390/foods9020198)
Supplement: Supplementary file 1 [file foods-09-00198-s001.pdf]

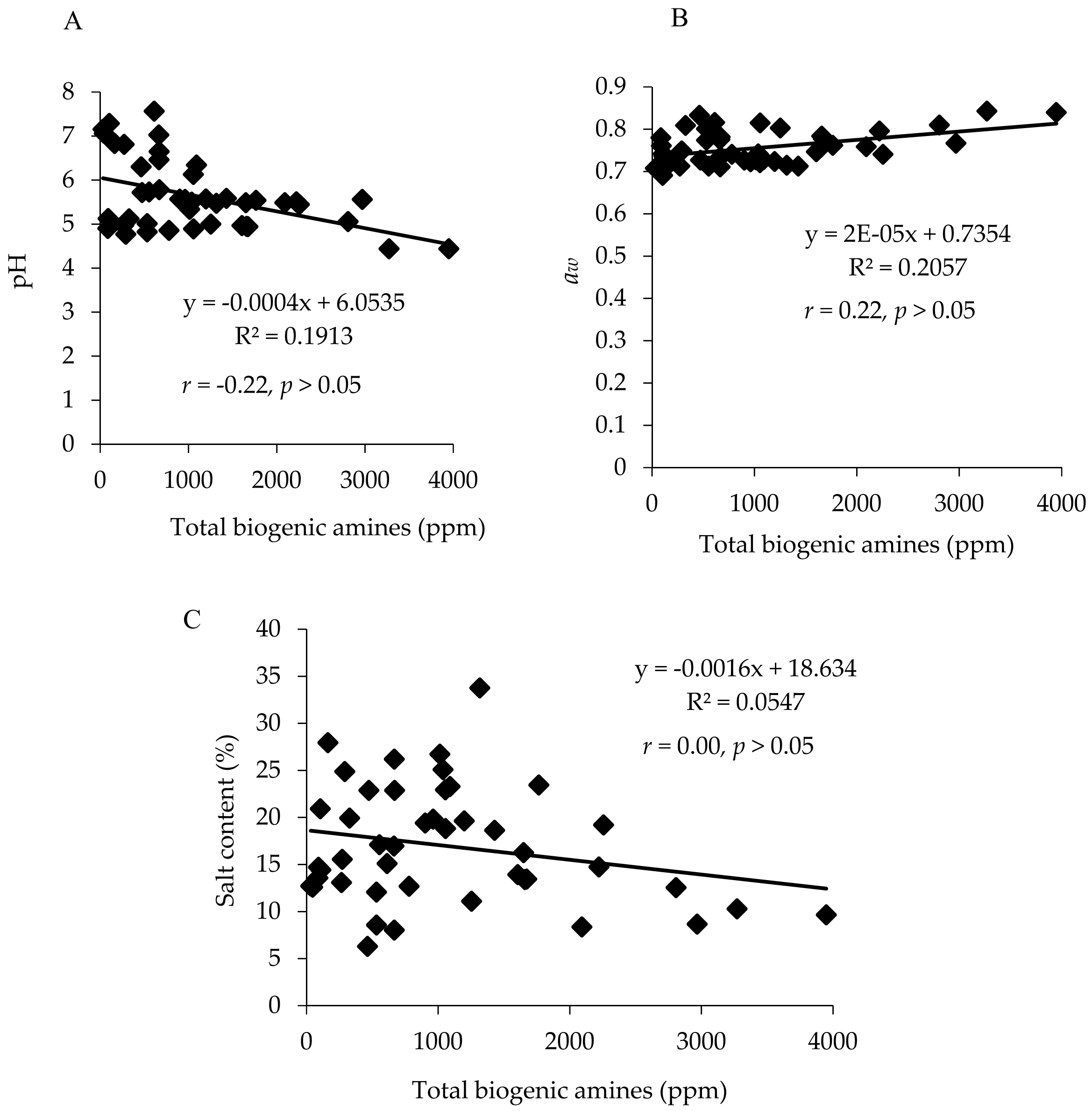

Figure S1. Linear fitting between total biogenic amines and physicochemical parameters, including pH (A), water activity (B), and salt content (C), in fermented fishery products (n = 42). Each dot indicates a data set obtained from a single sample.

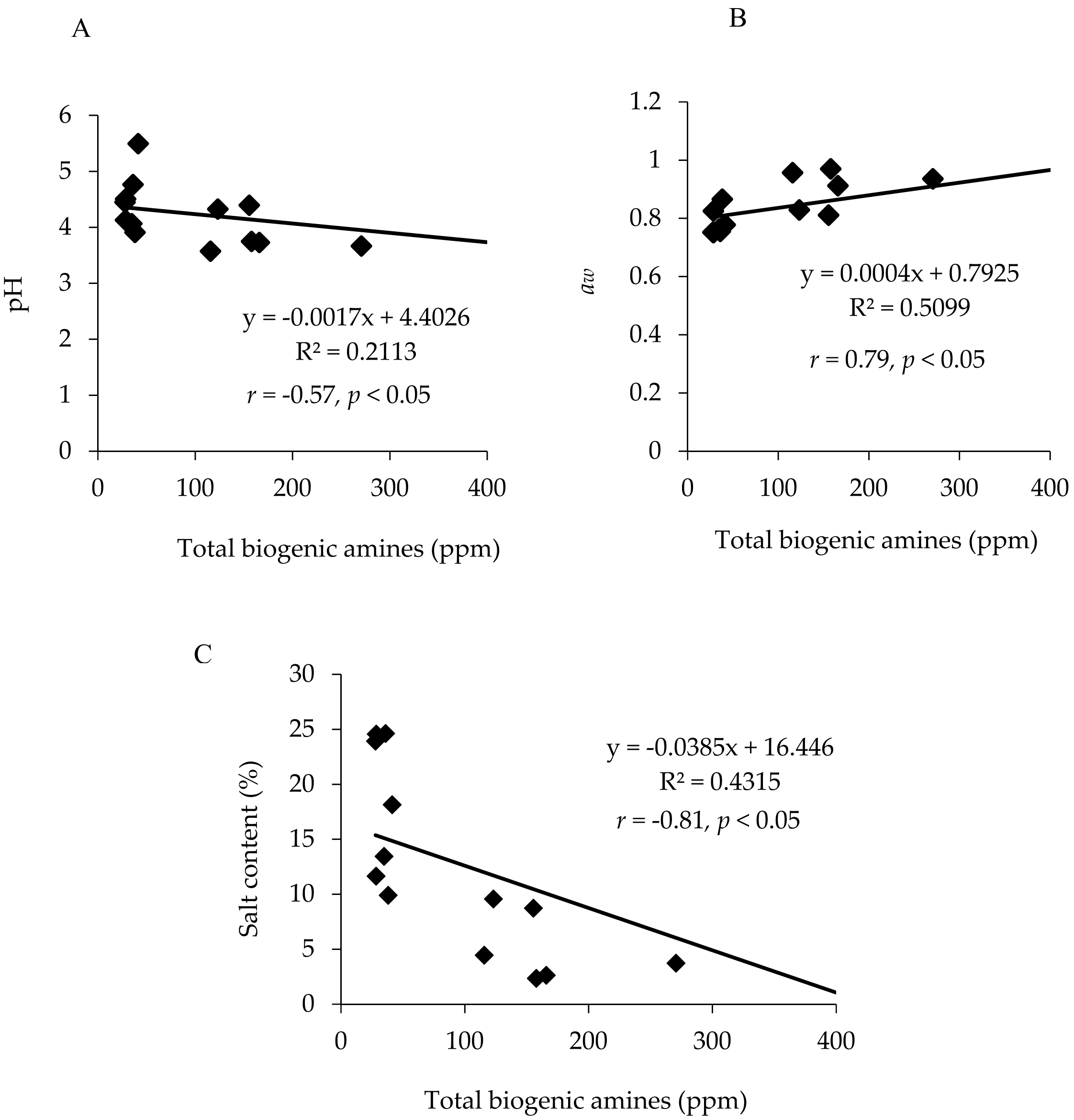

Figure S2. Linear fitting between total biogenic amines and physicochemical parameters, including pH (A), water activity (B), and salt content (C), in fermented vegetable products (n = 15). Each dot indicates a data set obtained from a single sample.

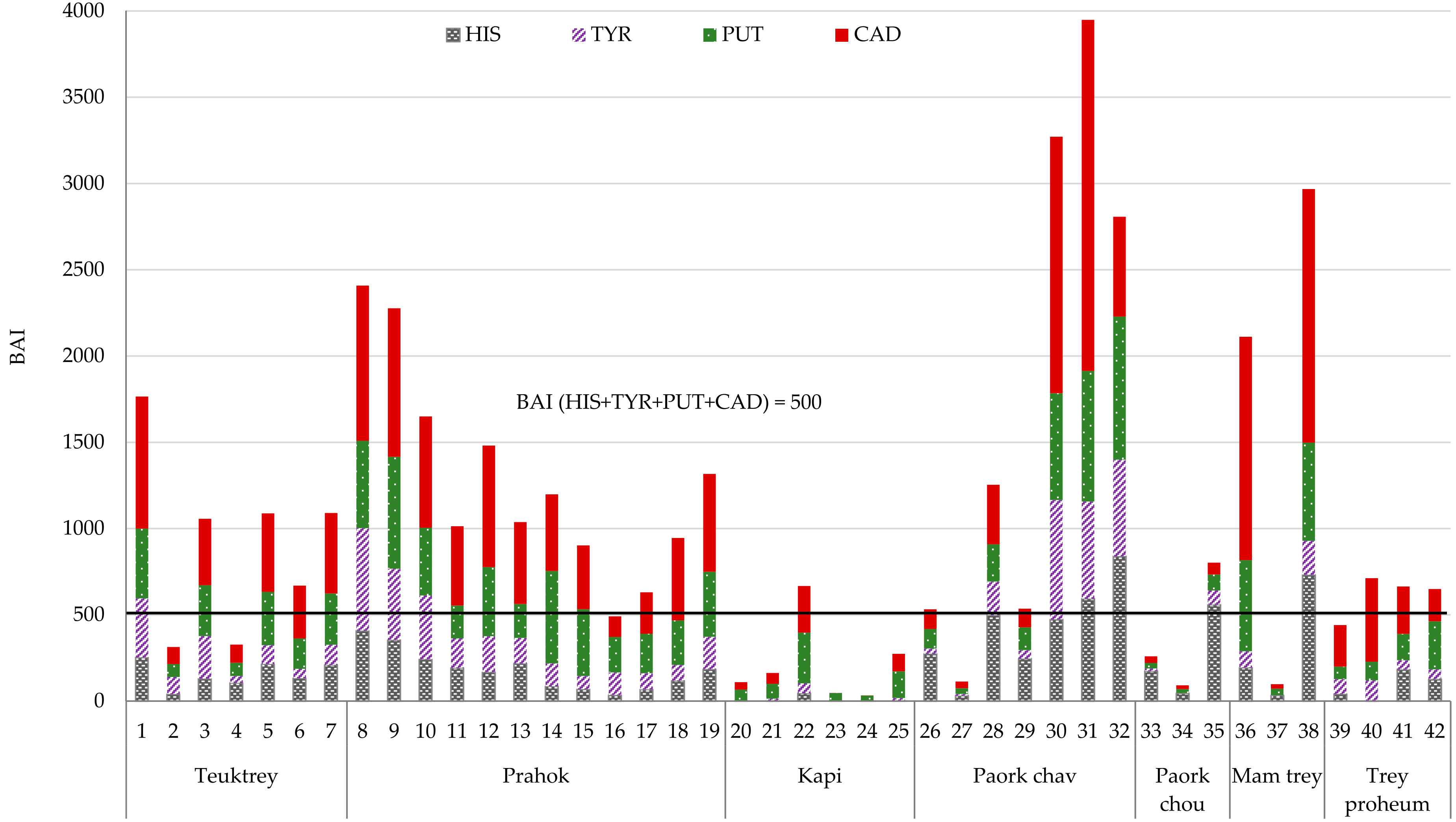

Figure S3. Biogenic amine index (BAI) evaluated for 42 samples of fermented fishery products. The bold horizontal line describes the limit value of 500 ppm, which is used to distinguish between fermented fishery products of good and poor hygienic quality.

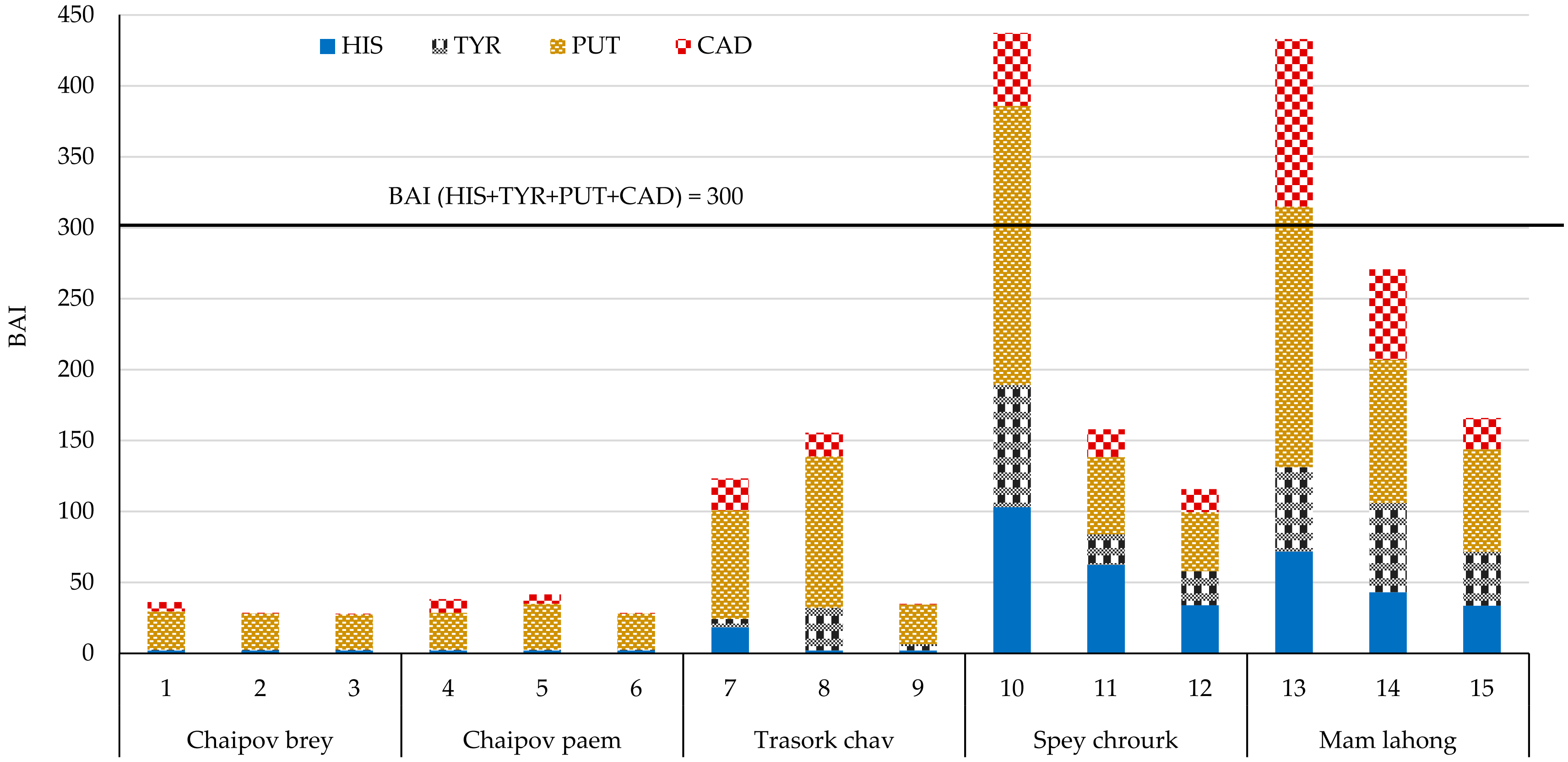

Figure S4. Biogenic amine index (BAI) evaluated for 15 samples of fermented vegetable products. The bold horizontal line describes the limit value of 300 ppm, which is used to distinguish between fermented vegetable products of good and poor hygienic quality.
